# Hormetic effect of panaxatriol saponins confers neuroprotection in PC12 cells and zebrafish through PI3K/AKT/mTOR and AMPK/SIRT1/FOXO3 pathways

**DOI:** 10.1038/srep41082

**Published:** 2017-01-23

**Authors:** Chao Zhang, Chuwen Li, Shenghui Chen, Zhiping Li, Lijuan Ma, Xuejing Jia, Kai Wang, Jiaolin Bao, Yeer Liang, Meiwan Chen, Peng Li, Huanxing Su, Simon Ming Yuen Lee, Kechun Liu, Jian-Bo Wan, Chengwei He

**Affiliations:** 1 State Key Laboratory of Quality Research in Chinese Medicine, Institute of Chinese Medical Sciences, University of Macau, Macao 999078, China; 2 Lee’s Pharmaceutical (Hong Kong) Ltd., Shatin, Hong Kong 999077, China; 3 Key Laboratory for Drug Screening Technology of Shandong Academy of Sciences, Shandong Provincial Key Laboratory for Biosensor, Biology Institute of Shandong Academy of Sciences, Jinan 250014, China

## Abstract

Hormesis is an adaptive response of living organisms to a moderate stress. However, its biomedical implication and molecular mechanisms remain to be intensively investigated. Panaxatriol saponins (PTS) is the major bioactive components extracted from *Panax notoginseng*, a widely used herbal medicine for cerebrovascular diseases. This study aims to examine the hormetic and neuroprotective effects of PTS in PC12 cells and zebrafish Parkinson’s disease (PD) models. Our results demonstrated that PTS stimulated PC12 cell growth by about 30% at low doses, while PTS at high doses inhibited cell growth, which is a typical hormetic effect. Moreover, we found that low dose PTS pretreatment significantly attenuated 6-OHDA-induced cytotoxicity and up-regulated PI3K/AKT/mTOR cell proliferation pathway and AMPK/SIRT1/FOXO3 cell survival pathway in PC12 cells. These results strongly suggested that neuroprotective effects of PTS may be attributable to the hormetic effect induced by PTS through activating adaptive response-related signaling pathways. Notably, low dose PTS could significantly prevent the 6-OHDA-induced dopaminergic neuron loss and improve the behavior movement deficiency in zebrafish, whereas relative high dose PTS exhibited neural toxicity, further supporting the hormetic and neuroprotective effects of PTS. This study indicates that PTS may have the potential in the development of future therapeutic medicines for PD.

Hormesis refers to a process in which exposure to a low dose of an environmental factor (physical, chemical or biological) that is damaging at higher doses induces an adaptive beneficial effect on the cell or organism^1^. Hormetic effects can be induced by various stimuli, such as radiation, toxins, natural compounds, pharmaceutical agents and endogenous agonists, in many biological models, including microbes, plants, invertebrates and mammals^2^,^3^, suggesting it is independent of biological model, endpoint measured, chemical class, and interindividual variability^4^. Hormesis is regarded as a set of evolutionarily conserved adaptive mechanisms to protect the living organisms from damage and enhance the survival in harsh environments^5^. Therefore, induction of hormesis was proposed to be a potential approach for prevention and treatment of diseases^6^.

Hormesis in nervous system, or named neurohormesis^7^, also has been observed in the studies on neuroprotection, neurite outgrowth, and pharmacology of Alzheimer’s disease, Parkinson’s disease (PD), anxiety, pain, seizures, stroke, behavioral disorders, etc.^8^. For instance, caloric restriction, a hormetic effector that exerts multiple beneficial effects, could increase the resistance of neurons to intracellular and extracellular stress and consequently improve the behavioral phenotype of neurological diseases in animal models^9^. Moreover, the effects of physical exercise on cognition and mood display a hormetic dose-response manner^10^ and these effects are closely related to the adult hippocampal neurogenesis^11^. A broad range of chemical agents, such as neurotrophic factors, antiexcitotoxins, steroids and phytochemicals that have been found to promote neuronal survival and neurite outgrowth, also generally exhibit biphasic hormetic dose-responses^12^. It is therefore reasonably speculated that the activation of hormetic mechanisms might be responsible, at least partially, for the neuroprotective effects of the biological and chemical factors.

*Panax notoginseng*, a highly valued medicinal herb, is efficacious in the prevention and treatment of cardio- and cerebro-vascular diseases and wound healing^13^. It can also significantly improve animal’s learning and memory, reduce neural cell apoptosis and infarct size after cerebral ischemia^14^. Panaxatriol saponins (PTS), the main components extracted from *Panax notoginseng*, have multiple bioactivities, including anti-platelet^15^, hepatoprotective^16^, anti-ischemia/reperfusion injury, etc.^17^. PTS have been reported to exhibit neuroprotective effects on oxygen-glucose deprivation-reperfusion induced PC12 cell death^18^ and 1-methyl-4-phenylpyridinium ion-induced neurotoxicity in mice^19^. However, the cellular and molecular mechanisms remain to be elucidated. In the present study, we firstly demonstrated that the neuroprotective activity of PTS in 6-hydroxydopamine (6-OHDA)-induced PD models in PC12 cells and zebrafish was attributable to PTS-elicited hormetic effect via regulating phosphoinositide 3-kinase (PI3K)/protein kinase B (AKT)/mammalian target of rapamycin (mTOR) and AMP-activated protein kinase (AMPK)/sirtuin-1 (SIRT1)/Forkhead box O3 (FOXO3) pathways.

## Results

### Hormetic effect of PTS protected PC12 cells against 6-OHDA-induced cell damage

**Figure 1:**
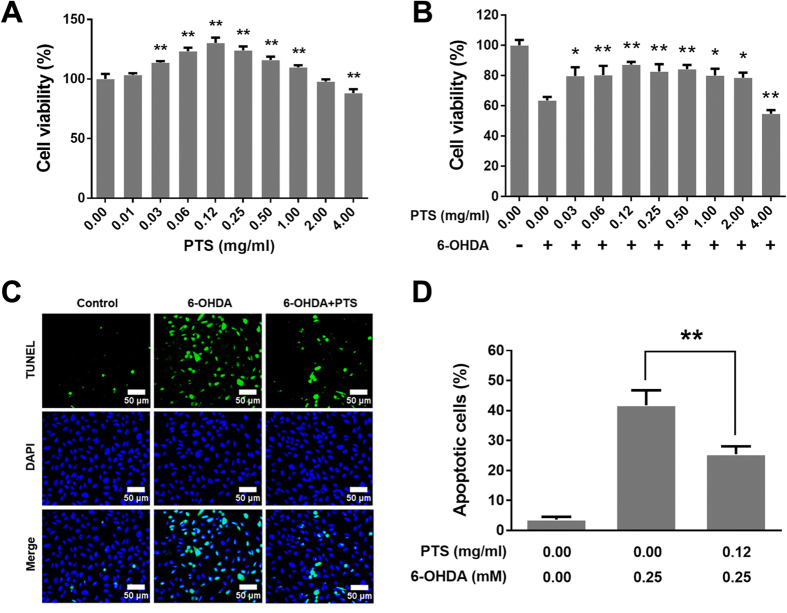
PTS induced hormetic effect in PC12 cells and protected PC12 cells against 6-OHDA-induced cell damage. The PC12 cells were treated with a wide range of concentrations of PTS for 24 h (**A**), and then incubated with or without 0.25 mM 6-OHDA for a further 24 h (**B**). The cell viability was determined by MTT assay. (**C**) PC12 cells were pretreated with 0.12 mg/ml PTS for 24 h and then treated with or without 0.25 mM 6-OHDA for 24 h and photographed using the InCell 2000 confocal microscope (20X objective) after Hoechst 33342 (blue) and TUNEL (green) staining. Scale bars represent 50 μm. (**D**) Quantification of apoptotic cells (**C**). Values represent the mean ± SD of at least three independent experiments. **P < 0.01, versus control group in (**A**); *P < 0.05, **P < 0.01, compared to 6-OHDA-treated alone groups in (**B** and **D**).

To examine the dose response, PC12 cells were treated with PTS at concentrations ranging from 0.01 to 4 mg/ml for 24 h. The cell viability of PTS was assessed by MTT assay. As shown in Fig. 1A, PTS at a concentration of 0.12 mg/ml increased cell proliferation by 30.3% and did not show cytotoxicity up to the concentration of 1 mg/ml. In contrast, treatment with PTS at a concentration of 4 mg/ml slightly reduced cell proliferation. This biphasic dose-response phenomenon was in line with the typical character of hormesis^1^,^20^.

We hypothesized that the hormesis induced by dose PTS could exhibit neuroprotective effects against 6-OHDA-induced neuronal cell death. To test this hypothesis, PC12 cells were treated with low concentrations of PTS (0.03–2 mg/ml) for 24 h and then incubated with or without 0.25 mM 6-OHDA for a further 24 h. As shown in Fig. 1B, PTS at low doses significantly protected PC12 cells from 6-OHDA-induced cell death. For example, 0.12 mg/ml PTS inhibited the cytotoxicity of 6-OHDA by 23.6%. However, co-treatment of relatively high dose of PTS (4 mg/ml) enhanced the cell growth inhibition of 6-OHDA in PC12 cells. Next, we determined whether PTS at low dose could protect PC12 cells against 6-OHDA-induced apoptosis. Results from TUNEL staining indicated that the apoptotic rates were decreased from 41.8% to 25.4% in the group of 6-OHDA (0.25 mM) used alone comparing to 6-OHDA plus PTS (0.12 mg/ml) (Fig. 1C and D). These results demonstrated that the hormetic effect of low dose PTS markedly suppressed 6-OHDA-induced neurotoxicity. The data also provided important information to discern the biomedical significance of hormesis.

### Low doses of PTS up-regulated PTEN/PI3K/AKT/mTOR and AMPK/SIRT1/FOXO3 pathways

**Figure 2:**
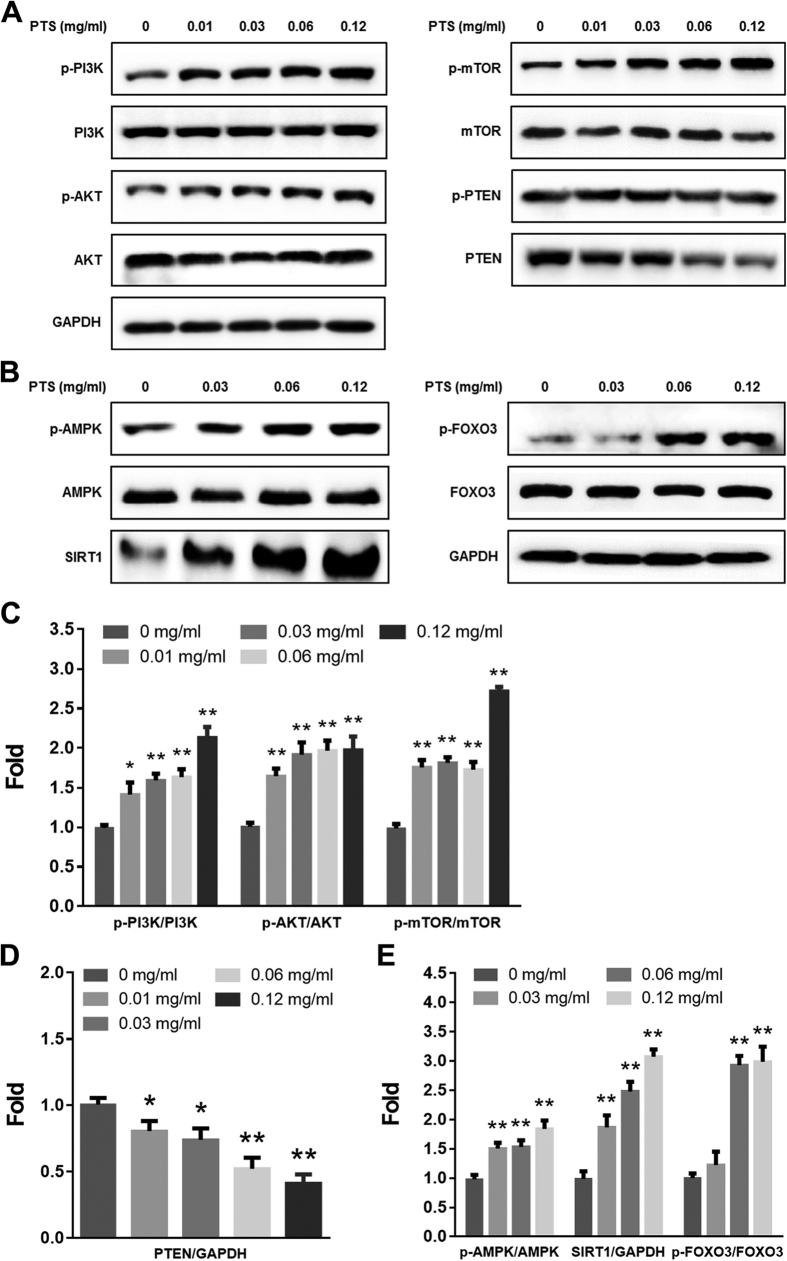
Effects of PTS on the expression levels of components of PTEN/PI3K/AKT/mTOR and AMPK/SIRT1/FOXO3 signaling pathways. PC12 cells were treated with varying concentrations of PTS for 24 h (**A,B**). Levels of total and phosphorylated proteins were determined by Western blot. (**C**–**E**) were densitometric analysis of (**A** and **B**) from three experiments. *P < 0.05 and **P < 0.01, versus control.

**Figure 3:**
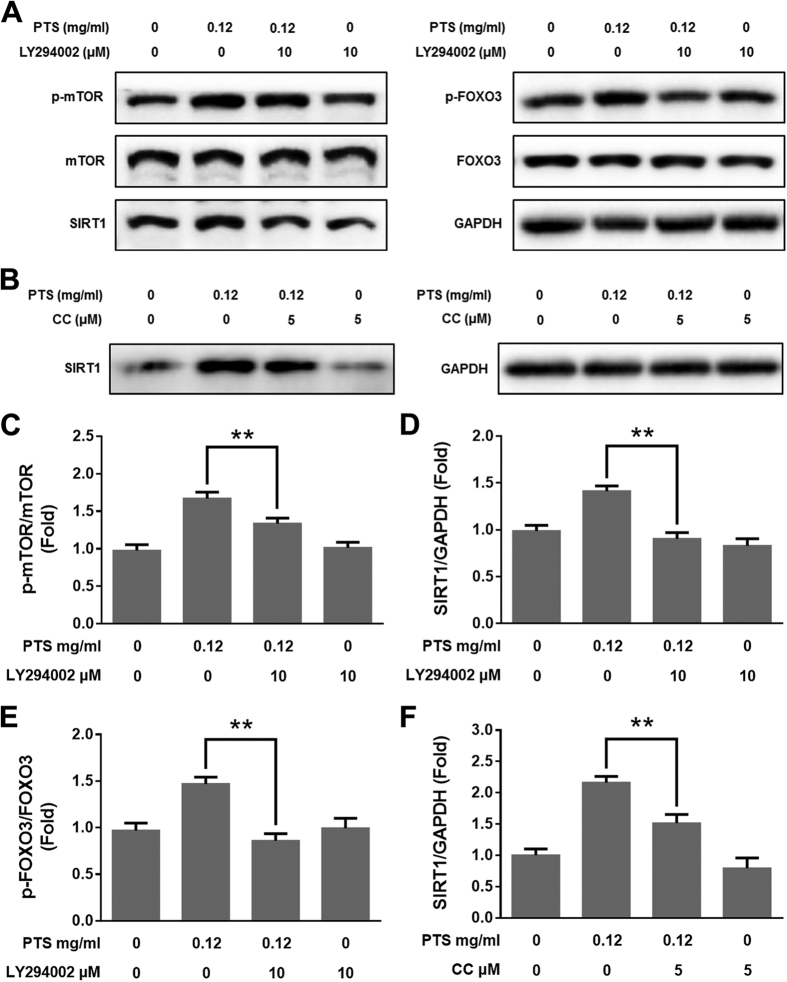
The roles of PI3K and AMPK in PTS-induced hormetic effect in PC12 cells. PC12 cells were exposed to 0.12 mg/ml PTS for 24 h with or without pretreatment of LY294002 (10 μM, 1 h) (**A**) or CC (5 μM, 1 h) (**B**). The protein levels of P-mTOR, mTOR, SIRT1, P-FOXO3 and FOXO3 were detected by Western blot. (**C–F**) were densitometric analysis of (**A** or **B**) from three experiments. **P < 0.01, compared to PTS-treated alone groups.

Since PI3K/AKT/mTOR pathway and AMPK/SIRT1/FOXO3 pathway play pivotal roles in cell proliferation and cell survival^21^,^22^, and adaptive oxidative response^23^,^24^. We hypothesized that PI3K/AKT/mTOR and AMPK/SIRT1/FOXO3 signaling pathways were involved in the hormetic effect induced by low doses of PTS. We examined the phosphorylated and total protein levels of PTEN, PI3K, AKT, mTOR, AMPK, SIRT1 and FOXO3 in PC12 cells treated with low doses of PTS by Western blotting assay. As shown in Fig. 2A and B, low doses of PTS increased the levels of p-PI3K, p-AKT, p-mTOR, p-AMPK and p-FOXO3, and the expression level of SIRT1 protein, and decreased the expression of PTEN protein in PC12 cells. PI3K/AKT inhibitor LY294002 significantly reversed the increased phosphorylation of mTOR and FOXO3, and protein level of SIRT1 (Fig. 3A). In addition, inhibition of AMPK by CC also significantly reversed the increased SIRT1 expression level (Fig. 3B). These results indicated that the hormetic effect of PTS at low doses on PC12 cells were through activating PTEN/PI3K/AKT/mTOR and AMPK/SIRT1/FOXO3 signaling pathways.

### Inhibition of PI3K/AKT/mTOR and AMPK/SIRT1/FOXO3 pathways attenuated the hormetic and neuroprotective effects of PTS

**Figure 4:**
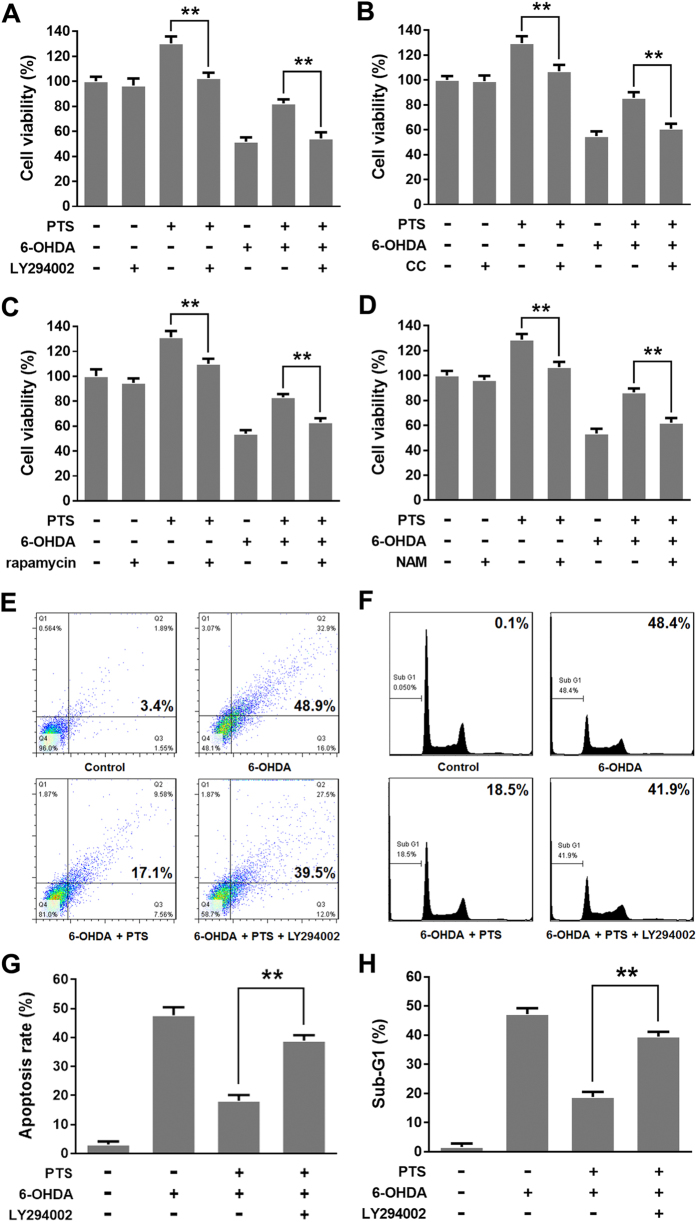
PI3K, AMPK, mTOR and SIRT1 inhibitors attenuated PTS-induced hormesis and neuroprotection in PC12 cells. PC12 cells were pre-incubated with or without 10 μM LY294002 (**A,E** and **F**), or 5 μM CC (**B**) for 1 h and then treated with 0.12 mg/ml PTS for 24 h; PC12 cells were treated with 0.12 mg/ml PTS for 24 h and then incubated with or without 100 nM rapamycin (**C**), or 10 mM NAM (**D**) for 1 h. PTS and inhibitor-treated cells were further treated with or without 0.25 mM 6-OHDA for 24 h. Cell viability was detected by MTT assay (**A–D**). The effect of PI3K inhibitor on the neuroprotective activity of PTS against 6-OHDA-induced apoptosis in PC12 cells was determined using Annexin V/PI staining (**E**) and subG1 peak analysis (**F**) by flow cytometry. (**G** and **H**) were quantified results of (**E** and **F**), respectively. Values represent the mean ± SD of at least three independent experiments. **P < 0.01, compared to PTS-treated alone groups or PTS+6-OHDA-treated groups in (**A–D**). **P < 0.01 versus PTS + 6-OHDA-treated groups in (**G** and **H**).

To further validate the role of PTEN/PI3K/AKT/mTOR cell proliferation pathway and AMPK/SIRT1/FOXO3 cell survival pathway in hormetic dose response of PTS, we tested whether the pathway inhibitors could reverse PTS-induced cell growth stimulation in PC12 cells. The MTT colorimetric assay revealed that co-treatment of PTS and 10 μM LY294002 (Fig. 4A), 5 μM CC (Fig. 4B), 100 nM rapamycin (Fig. 4C), or 10 mM NAM (Fig. 4D) partially abolished the growth stimulation by low dose of PTS in PC12 cells comparing to PTS treated alone, suggesting that PI3K/AKT/mTOR and AMPK/SIRT1/FOXO3 pathways are involved, at least partially, in the hormetic effect of PTS at low doses on PC12 cells.

We further investigated whether PI3K/AKT/mTOR and AMPK/SIRT1/FOXO3 pathways participates in the neuroprotective effect of low dose PTS in PC12 cells. The MTT colorimetric assay revealed that LY294002 (Fig. 4A), CC (Fig. 4B), rapamycin (Fig. 4C) or NAM (Fig. 4D) partially abolished the inhibitory effect of low dose PTS on 6-OHDA-induced cell death comparing to co-treatment of PTS and 6-OHDA. We further assessed if the neuroprotective effect of PTS against 6-OHDA-induced apoptosis was affected by PI3K/AKT pathway inhibitor in PC12 cells. Results from Annexin V/PI staining (Fig. 4E) and sub-G1 cell analysis (Fig. 4F) indicated that the apoptotic rate was decreased in PC12 cells co-treated with 6-OHDA and PTS comparing to the 6-OHDA treatment alone. However, the inhibitory effect of PTS on apoptosis-induction by 6-OHDA was significantly abolished by LY294002. (Fig. 4E and F). These results demonstrated that PI3K/AKT/mTOR and AMPK/SIRT1/FOXO3 pathways were involved, at least partially, in the neuroprotective effect of low dose PTS on PC12 cells.

### Low doses of PTS prevented 6-OHDA-induced DA neuronal loss in zebrafish

**Figure 5:**
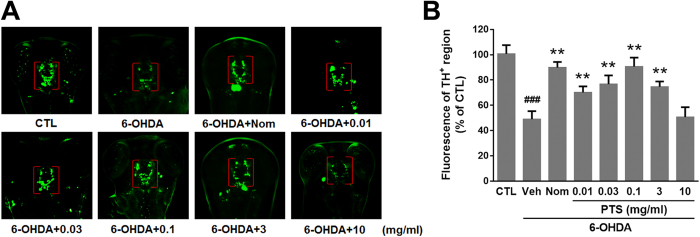
The effect of PTS on 6-OHDA-induced dopaminergic (DA) neuron loss in zebrafish. Zebrafish embryos at one day post fertilization were exposed to indicated concentrations of PTS or nomifensine (Nom, used as a positive control) in the presence or absence of 0.25 mM 6-OHDA for 48 h. Then larvae were fixed for whole mount immunostaining with antibody against tyrosine hydroxylase (TH). (**A**) Representative morphology of DA neurons in the zebrafish brain. TH^+^ neurons in the diencephalic region are within brackets. (**B**) Statistical analysis of TH^+^ neurons in each group of ten fish. Values represent the mean ± SD of at least three independent experiments. Data are expressed as a percentage of the control group. ^###^*P* < 0.01 versus control (CTL) group, **P < 0.01 versus 6-OHDA-treated group.

To investigate the neuroprotective effect of PTS *in vivo*, anti-TH whole-mount immunofluorescent staining was used to examine DA neurons in 6-OHDA-treated zebrafish larvae. As shown in Fig. 5A and B, 48 h exposure of 0.25 mM 6-OHDA resulted in about 50% loss of DA neurons in the diencephalon of zebrafish (indicated by red brackets). Importantly, co-treatment with low dose PTS (0.01, 0.03 and 0.1 mg/ml) could significantly ameliorate, even almost completely reverse the DA neuron loss induced by 6-OHDA, which was similar to the activity of positive control agent Nom, an inhibitor of dopamine transporter. However, high dose PTS (10 mg/ml) failed to protect against 6-OHDA-induced DA neuron loss. These results indicated that PTS at relatively low doses exhibited strong protective effect against 6-OHDA-induced DA neuron death in zebrafish, and this neuroprotection was in a hormetic dose response manner.

### Low doses of PTS suppressed 6-OHDA-induced deficits in the locomotive behavior of zebrafish

**Figure 6:**
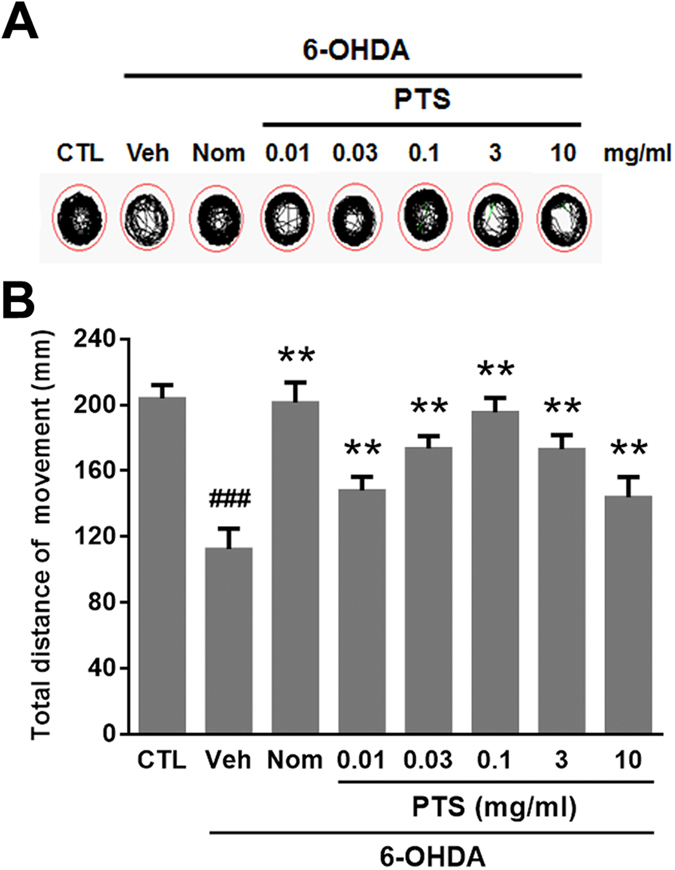
The effect of PTS on 6-OHDA-induced deficits in the locomotor behavior of zebrafish. Zebrafish at 3 day post fertilization were exposed to indicated concentrations of PTS or Nom with or without 0.25 mM 6-OHDA for 4 days. Then zebrafish were collected and the locomotor activity of each group was monitored using the Viewpoint Zebrabox system; total distance traveled in 10 min was calculated. (**A**) Representative patterns of zebrafish locomotion traced from control and different treatment groups. (**B**) Statistical analysis of total distance moved of different treatment groups, eight fish larvae/group from three independent experiments. ^###^*P* < 0.01 versus CTL group, **P < 0.01 versus 6-OHDA-treated group.

In zebrafish larvae, injury of DA neurons affects mobility. As shown in Fig. 6, compared with the control group, 6-OHDA markedly reduced the movement distance and altered the swimming behavior of zebrafish larvae (from 204 to 112 mm), whereas Nom alleviated this deficit (201 mm). Under the same conditions, 0.01, 0.03 and 0.1 mg/ml of PTS inhibited 6-OHDA-induced movement decreases in a concentration-dependent manner (148, 174 and 195 mm, respectively). However, high dose PTS (10 mg/ml) only slightly inhibited the 6-OHDA-induced reductions in zebrafish total movement distance (141 mm).

## Discussion

Hormesis is one of the adaptive mechanisms for living organisms to survive and reproduce in harsh competitive environments. Under this scope, mild (low dose) challenging factors, such as radiations and chemicals are fundamental stimulators that provide selective pressure on organisms to maintain an adequate survival capacity. Therefore, the concept of hormesis may have vast and deep impact on biomedical sciences, such as the areas of aging, longevity and neuroscience^25^,^26^,^27^. Numerous phytochemicals (e.g., resveratrol, curcumins, ginsenosides, naringin, protocatechuic acid, chrysin, epigallocatechin-gallate, kaempferol, etc.) have shown therapeutic potential against neurodegenerative diseases^28^,^29^. However, the underlying cellular and molecular mechanisms are largely unknown. These phytochemicals have often been found to exhibit biphasic hormetic dose responses^12^ and activate adaptive cellular stress pathways^30^. In the current study, we provided direct evidence to indicate that hormetic effect and the related signaling pathways were responsible for the neuroprotective activity of PTS, a class of saponins extracted from *Panax notoginseng*.

The characteristic of hormesis is a biphasic dose-response with a low dose stimulatory/beneficial effect (improved function, increased resistance to damage and disease) and a high dose inhibitory/toxic effect (dysfunction, molecular damage, or even death)^31^, resulting in either a J-shaped or an inverted U-shaped dose-response. Hormesis response is usually modest, with the magnitude of the maximum stimulatory responses is about 30–60% above that of the control response, and the width of the stimulatory dosage is typically within 100-fold of the threshold value^32^. We found that PTS induced a typical hormetic dose-response in PC12 cells, a rat pheochromocytoma cell line with typical neuron features, and has been widely introduced as a neuronal cell *in vitro* model^33^. Our results showed that the effect of PTS on PC12 cell viability tested by MTT displayed an inverted-U-shaped dose-response curve, with the highest stimulatory rate of cell growth of 30% by 0.12 mg/ml PTS, and with a stimulatory dosage as low as about 100-fold below the threshold value (Fig. 1A). These results indicated that PTS induced a typical hormetic response in PC12 cells.

To test whether PTS exhibit neuroprotection *in vitro*, we examine the effect of PTS on 6-OHDA-induced cytotoxicity in PC12 cells. 6-OHDA, a dopamine analog that could cause excitotoxic damage in DA neuronal death, is commonly used to generate experimental cellular and animal models of PD^34^. The results indicated that PTS at the concentrations ranging from 0.03 to 2 mg/ml significantly ameliorated the cell growth inhibition and apoptosis induced by 6-OHDA in PC12 cells (Fig. 1B and C), which was in line with the previous reports that PTS protected PC12 cells against oxygen-glucose deprivation reperfusion- and 1-methyl-4-phenylpyridinium ion-induced cell damage^18^,^19^. However, relatively high concentration of PTS (4 mg/ml) did not show neuroprotective activity but enhanced the cytotoxicity of 6-OHDA (Fig. 1B). Notably, the doses of PTS that elicited hormetic effects were identical to that of exerting neuroprotective activity, suggesting that PTS-induced hormetic effect may contribute to the neuroprotection against 6-OHDA in PC12 cells.

The neuroprotective effect of PTS was confirmed in a 6-OHDA-induced zebrafish PD model, which is a widely used powerful tool for drug discovery for neurodegenerative diseases. The results of TH immunostaining, which is specific for DA neurons, demonstrated that relatively low doses (0.01 to 3 mg/ml) of PTS significantly reduced the 6-OHDA-caused DA neuron loss in the brain of zebrafish (Fig. 5). In addition, results of locomotor behavior showed that PTS at the same range of doses remarkably reduced the 6-OHDA-caused decrease in the movement distance of zebrafish (Fig. 6). Markedly, the neuroprotective activity of PTS against both DA neuron loss and locomotion deficit was comparable to that of the positive control agent Nom, a dopamine transporter inhibitor. However, high dose (10 mg/ml) of PTS only showed slight protective effects in zebrafish (Figs 5 and 6), further substantiated the hormetic dose response of PTS in neuroprotection. Similar results were observed in our previous report showed that low dose camptothecin (CPT), a topoisomerase I inhibitor, exhibited hormetic and neuroprotective effects, while high dose CPT killed the cells^35^. Schisandrin B, an active ingredient isolated from *Fructus Schisandrae*, has been shown to prevent age-related neurodegenerative diseases through inducing hormesis^36^. Moreover, induction of mild endoplasmic reticulum stress and autophagy, which were thought to be hormetic mechanisms, could inhibit neuronal cell death in drosophila and mouse models of PD^37^,^38^. Taking together, these findings strongly suggested that induction of hormetic effect might contribute, at least partially, to the neuroprotective activity of many phytochemicals.

To survive in a challenging environment, organisms have developed complex hormetic mechanisms to protect against various hazardous factors. These mechanisms involve a broad range of stress response proteins and pathways, such as membrane transporters, protein chaperones, antioxidant enzymes, growth factors, and signaling pathways and transcriptional factors that regulate the aforementioned proteins^39^. However, hormetic mechanisms may vary among different stimuli on certain cell types^40^. PI3K/AKT is a major pathway mediating neuronal survival by promoting cell proliferation and inhibiting apoptosis^41^, and emerging as a potential therapeutic targets for neurodegenerative diseases^42^. AKT, or protein kinase B, signaling cascade is activated by receptor-mediated production of phosphatidylinositol-(3,4,5)- trisphosphates (PIP3) by PI3K, and is inhibited by the tumor-suppressor PTEN through dephosphorylating PIP3 back to PIP2. Phosphorylation of AKT by mTOR Complex 1 (mTORC1) leads to a full enzymatic activity, which triggers expression of numerous proteins involving in regulating cell growth and cell death^43^. mTOR is one of the direct target proteins of AKT, by which mTOR is phosphorylated/activated, leading to regulations of multiple cellular functions, such as cell death and survival, protein synthesis, and autophagy^44^. In the present study, we found that PTS at low doses significantly increased the levels of p-PI3K, p-AKT and p-mTOR, while decreased total protein level of PTEN in PC12 cells (Fig. 2A). Inhibition of the PI3K/AKT/mTOR pathway with LY294002 (a PI3K/AKT pathway inhibitor) and rapamycin (an mTOR inhibitor) reversed the proliferation-promoting effect of low dose PTS in PC12 cells (Fig. 4A and C). Importantly, the protective effect of low dose PTS against 6-OHDA-induced cell growth inhibition was significantly attenuated by PI3K and mTOR inhibitors (Fig. 4A and C), and the inhibition of PTS on 6-OHDA-induced apoptosis was also abolished by LY294002 (Fig. 4E and F). Additionally, LY294002 could significantly reduce the level of p-mTOR in the presence of PTS (Fig. 3A). These results demonstrated that the hormetic and neuroprotective effects of PTS was through, at least partially, the activation of PTEN/PI3K/AKT/mTOR pathway, which were in line with previous reports on hormesis in PC12 cells induced by low dose CPT^35^ or Z-ligustilide^45^.

AMPK acts as a key regulator of energy metabolic homeostasis and is important in adaptive response processes^46^. Both AMPK and sirtuins are regarded as antiaging or longevity proteins^46^,^47^. There is a positive feedback loop interaction between AMPK and SIRT1, one of the most extensively researched sirtuins. Evidence suggests that the activation of AMPK-SIRT1 pathway has been associated with longevity in a number of species^24^. SIRT1 plays key role in adaptive responses of cells to a variety of oxidative stressors by deacetylating several FOXO members, which are known to be crucial regulators of apoptosis and oxidative stress resistance^48^,^49^. FOXO3 is one of the FOXO proteins and its transcriptional activity is modulated by both AMPK and SIRT1^50^. Our data showed that low dose PTS treatment significantly increased the levels of p-AMPK and p-FOXO3, and the expression level of SIRT1 by Western blot analysis (Fig. 2B). The induction of SIRT1 expression by low dose PTS could be attenuated by AMPK inhibitor CC (Fig. 3B), and CC or NAM (a SIRT1 inhibitor) abolished the proliferation-promoting effect of PTS and the protective effect of low dose PTS against 6-OHDA-induced cell death (Fig. 4B and D), suggesting that AMPK/SIRT1/FOXO3 pathway is also involved in the hormetic and neuroprotective effects of low dose PTS on PC12 cells. It was reported that resveratrol prevented oxidative stress-induced proliferative dysfunction and senescence by activating the AMPK-SIRT1-FOXO3 pathway in human primary keratinocytes^24^, and resveratrol commonly displays hormesis^51^, further supporting our speculation.

Studies indicated that the AKT could phosphorylate FOXO proteins, resulting in pro-survival and anti-apoptosis activities^52^,^53^. For example, the white wine component, *n*-tyrosol pretreatment could confer cardioprotection against an ischemic insult in rat model of myocardial infarction through the activation of AKT/FOXO3/SIRT1 pathway^54^. Dong *et al*. reported that magnolol protected SH-SY5Y cells against acrolein-induced neural cell damage through regulating PI3K/AKT/FOXO1 signaling pathway^55^. In our results, the PTS-upregulated SIRT1 expression and phosphorylation of FOXO3 were attenuated by the pretreatment of LY294002 (Fig. 3A), confirming the crosstalk between AKT and SIRT1/FOXO3.

**Figure 7:**
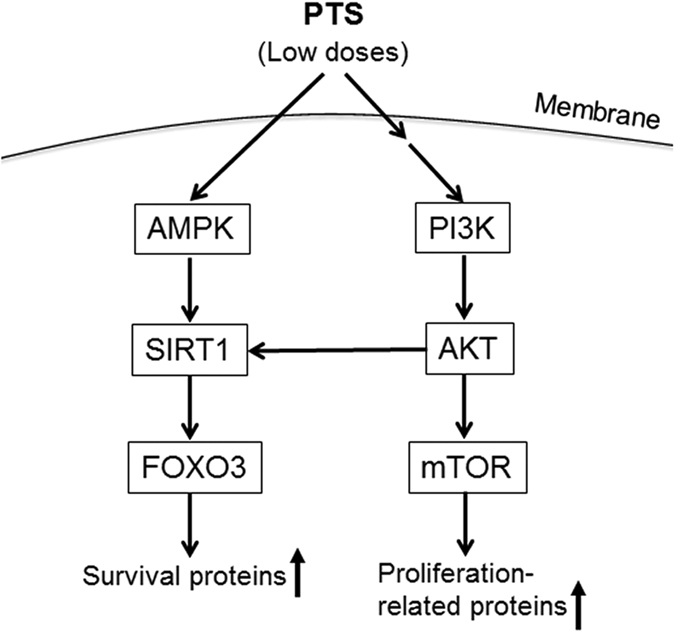
A schematic model of upregulated AMPK/SIRT1/FOXO3 cell survival pathway and PI3K/AKT/mTOR cell proliferation pathway by low doses of PTS in PC12 cells.

In summary, we demonstrated that low dose PTS induced hormetic effect and thereby neuroprotection against 6-OHDA-induced cell growth inhibition and apoptosis through activating PI3K/AKT/mTOR cell proliferation pathway and AMPK/SIRT1/FOXO3 cell survival pathway (as summarized in Fig. 7) in PC12 cells. In addition, PTS remarkably protected zebrafish against 6-OHDA-induced loss of DA neurons and reduction in locomotor movement in a hormetic dose response manner. Our findings provided direct experimental evidences that stimulation of hormetic adaptive responses in neuronal cells could not only be a general mechanism for the neuroprotective activity of numerous phytochemicals without specific targets, but also a new approach for the prevention and treatment of neurodegenerative diseases. Moreover, we confirmed that PTS had the potential to be developed as a therapeutic or preventive agent for neurodegenerative diseases.

## Materials and Methods

### Chemicals and reagents

PTS was isolated^56^,^57^,^58^ and kindly provided by Dr. Jian-Bo Wan (University of Macau). The HPLC profiles of five reference compounds *i.e.* notoginsenoside R_1_, and ginsenosides Rg_1_, Re, Rb_1_, Rd (Supplementary Fig. S1), and PTS (Supplementary Fig. S2) were determined using a Waters e2695 HPLC system, equipped with a Waters 2996 Photodiode Array Detector. All reference compounds including notoginsenoside R_1_, ginsenosides Rg_1_, Re, Rb_1_, and Rd, were purchased from National Institute for the Control of Pharmaceutical and Biological Products (Beijing, PR, China). F-12K medium, penicillin-streptomycin (PS), phosphate buffered saline (PBS) were supplied by Gibco (Maryland, USA). Fetal bovine serum (FBS) and horse serum (HS) were obtained from Invitrogen (Carlsbad, CA, USA). 3-(4,5-dimethylthiazol-2-yl)-2,5-diphenyltetrazolium bromide (MTT) was obtained from Molecular Probes (Eugene, OR, USA). Primary antibodies against p-PI3K, PI3K, p-AKT, AKT, p-mTOR, mTOR, p- phosphatase with tensin homology (PTEN), PTEN, p-AMPK, AMPK, SIRT1, p-FOXO3, FOXO3 and GAPDH, and secondary antibodies were purchased from Cell Signaling Technology (Danvers, MA, USA) or Proteintech (Chicago, IL, USA). Annexin V-fluorescein isothiocyanate (FITC)/propidium iodide (PI) apoptosis detection kit, Hoechst 33342 staining kit, cell cycle and apoptosis analysis kit, rapamycin, nicotinamide (NAM), LY294002, and the terminal deoxynucleotidyl transferase-mediated dUTP nick end-labeling (TUNEL) cell apoptosis detection kits were purchased from Beyotime (Nanjing, Jiangsu, China). Compound C (CC) was obtained from Calbiochem (Billerica, MA, USA). 6-OHDA and nomifensine (Nom) were supplied by Sigma-Aldrich Co (St. Louis, MO, USA). The enhanced chemiluminescence (ECL) detection kit was purchased from BD Biosciences (Bedford, MA, USA). All other chemicals of analytical grade were purchased from local sources.

### Cell culture and drug treatments

PC12, a rat adrenal pheochromocytoma cell line, was obtained from American Type Culture Collection (Manassas, VA, USA). Cells were cultured in ATCC-formulated F-12K medium supplemented with 15% heat-inactivated HS, 2.5% FBS, and 1% antibiotics (100 units/mL PS), in a humidified atmosphere of 5% CO_2_ at 37 °C. The culture medium was changed every two days. For all *in vitro* assays, the working solutions of PTS were freshly dissolved and diluted in the basal medium.

### Cell viability assay

Cell viability was evaluated by MTT colorimetric assay^59^. Briefly, PC12 cells (6 × 10^3^ cells/well) were treated with a wide range of concentrations of PTS for 24 h in 96-well plates. To test the neuroprotective effect of PTS at low doses against 6-OHDA-induced cell damage, PC12 cells were pretreated with indicated concentrations of PTS for 24 h prior to the treatment of 0.25 mM 6-OHDA for another 24 h. The treated cells were then incubated in 0.5 mg/ml MTT solution for another 4 h at 37 °C. The supernatants were replaced with DMSO to dissolve the violet formazan crystals. The absorbance at 570 nm was determined using a microplate reader (BioTek, Winooski, VT, USA). The relative viability of treated cells was expressed as percentage of control untreated cells.

### TUNEL staining

We performed TUNEL method to label 3′-end of fragmented DNA of the apoptotic PC12 cells. Cells were fixed with 4% paraformaldehyde, washed with PBS, and incubated with 0.1% TritonX-100 for 2 min on ice followed by TUNEL staining according to the manufacturer’s instructions. The FITC-labeled TUNEL-positive cells were imaged using the InCell 2000 confocal microscope (GE Biosciences, Piscataway, NJ, USA). The cells with green fluorescence were described as apoptotic cells. Quantitative analysis of apoptotic cells content among groups was carried out using the software modules supplied with the InCell 2000.

### Annexin V-FITC/PI staining

Annexin V-FITC/PI double staining was carried out to determine apoptosis in PC12 cells by flow cytometry (FCM). The cells were harvested and washed with PBS, incubated in binding buffer containing Annexin V-FITC and PI for 15 min at 37 °C in the dark. Cells were then analyzed using FCM (FACS Canto^TM^, BD, CA, USA). The number of apoptotic cells per sample was counted using FlowJo software version 7.6.1 (Ashland, OR, USA).

### Flow cytometric analysis for measurement of sub-G1 phase

For sub-G1 DNA content analysis, which is a characteristic of apoptosis, the treated cells were collected and washed with PBS followed by fixation with ice-cold 70% ethanol and placed at -20 °C for 24 h, and then incubated with PI for 15 min in the dark. Samples were analyzed using FCM (FACS Canto^TM^, BD, CA, USA). The percentage of sub-G1 DNA content per sample was counted using FlowJo software version 7.6.1.

### Western blotting

PC12 cells were collected and lysed by RIPA buffer. Protein concentration was determined by the BCA protein assay kit (Thermo Scientific, Rockford, IL, USA). Equivalent amounts of proteins from each group were subjected to SDS-PAGE gel electrophoresis, and transferred onto polyvinylidene fluoride membranes (Bio-Rad, Philadelphia, PA, USA). After being blocked with 5% non-fat milk in Tris-buffered saline buffer, the membranes were incubated with primary antibodies (1:1000) at 4 °C overnight, followed by incubation with the corresponding secondary antibodies (1:5000). Protein bands were visualized with an ECL advanced Western blotting detection kit. The density of the bands was quantified by the Image Lab Software (Bio-Rad, Hercules, CA, USA).

### Inhibitor treatment

To clarify the roles of signaling pathways in low doses of PTS-induced hormetic effects in PC12 cells, the cells were pretreated with the following inhibitors individually before the treatment of PTS (0.12 mg/ml): 10 μM LY294002 (PI3K/AKT pathway inhibitor), 5 μM CC (AMPK inhibitor), 100 nM rapamycin (mTOR inhibitor), or 10 mM NAM (SIRT1 inhibitor). The cells were then subjected to the measurement of cell viability of PC12 cells and hormesis-related protein levels by MTT assay and Western blotting. To analyze the roles of signaling pathways in low concentrations of PTS-triggered neuroprotective effects in PC12 cells, the cells were treated with PTS (0.12 mg/ml) for 24 h followed by incubation with rapamycin or NAM for 1 h. In addition, PC12 cells were pre-incubated with LY294002 or CC for 1 h and then treated with PTS (0.12 mg/ml) for 24 h. Drug-treated cells were further treated with 0.25 mM 6-OHDA for 24 h. The cells were then subjected to the determination of cell viability by MTT assay, and apoptosis by Annexin V-FITC/PI staining and sub-G1 DNA content analysis.

### Anti-tyrosine hydroxylase (TH) whole-mount immunostaining

The wild-type AB strain of zebrafish was used in this study and maintained as described previously^29^. Zebrafish embryos at 1 day post fertilization (dpf) were exposed to 0.25 mM 6-OHDA in the presence or absence of indicated concentrations of PTS or Nom (used as a positive control) for 48 h. Then zebrafish larvae were fixed with 4% paraformaldehyde in PBS for 30 min, rinsed and stored at −20 °C in absolute MtOH. Whole-mount immunostaining and semi-quantification of TH-positive dopaminergic (DA) neurons were performed as previously described with slight modification^29^. The results are expressed as a percentage of the area of TH^+^ cells in untreated normal control group. All experiments and animal care procedures in this study were performed according to the Guide to Animal Use and Care of the University of Macau (UM) and were approved by the ethics committee of UM.

### Zebrafish locomotion assay

Zebrafish larvae at 3 dpf were treated with indicated concentrations of PTS or Nom in the absence or in the presence of 0.25 mM 6-OHDA for 4 days, and then zebrafish at 7 dpf were transferred into 96-well plates (1 fish/well). Zebrafish behavior was monitored by a digital video tracking system (Viewpoint, ZebraLab, LifeSciences). The total distance moved and swimming pattern were recorded in a 10 min long session. The larvae were allowed to habituate to the environment of the system for 30 min before the start of the data acquisition.

### Statistical analysis

All the data were given as means ± standard deviation of three independent experiments. Statistical analysis was done by One-way ANOVA with Tukey post hoc analysis using GraphPad Prism statistical software (GraphPad Software, San Diego, CA, USA). Statistical significance was accepted at the level of *P* < 0.05.

## Additional Information

**How to cite this article**: Zhang, C. *et al*. Hormetic effect of panaxatriol saponins confers neuroprotection in PC12 cells and zebrafish through PI3K/AKT/mTOR and AMPK/SIRT1/FOXO3 pathways. *Sci. Rep.* **7**, 41082; doi: 10.1038/srep41082 (2017).

**Publisher's note:** Springer Nature remains neutral with regard to jurisdictional claims in published maps and institutional affiliations.

## Supplementary Material

Supplementary Dataset 1
