# Supplementary material for: Hormetic effect of panaxatriol saponins confers neuroprotection in PC12 cells and zebrafish through PI3K/AKT/mTOR and AMPK/SIRT1/FOXO3 pathways
Source: Sci Rep. 2017 Jan 23;7:41082. doi: 10.1038/srep41082 (PMC5253660; doi:10.1038/srep41082)
Supplement: Supplementary Dataset 1 [file srep41082-s1.doc]

**Hormetic effect of** **panaxatriol saponins confers neuroprotection in PC12 cells and zebrafish through PI3K/AKT/mTOR and AMPK/SIRT1/FOXO3 pathways**

Chao Zhang^1,+^, Chuwen Li^1,+^, Shenghui Chen^1,2^, Zhiping Li^3^, Lijuan Ma^1^, Xuejing Jia^1^, Kai Wang^1^, Jiaolin Bao^1^, Yeer Liang^1^, Meiwan Chen^1^, Peng Li^1^, Huanxing Su^1^, Simon Ming Yuen Lee^1^, Kechun Liu^3^, Jian-Bo Wan^1,*^, Chengwei He^1,*^

**Supplementary Table 1**

**Calibration curves**

| Analytes | RT(min) | Calibration curve | r^2^ |
| --- | --- | --- | --- |
| R1 | 25.011 | y=3.9E-7x+0.0048 | 0.9999 |
| Rg1 | 34.371 | y=3.2E-7x+0.0020 | 1.0000 |
| Re | 35.065 | y=4.0E-7x-0.0009 | 1.0000 |
| Rb1 | 45.086 | y=4.7E-7x-0.0076 | 0.9937 |
| Rd | 48.210 | y=3.3E-7x+0.0003 | 1.0000 |


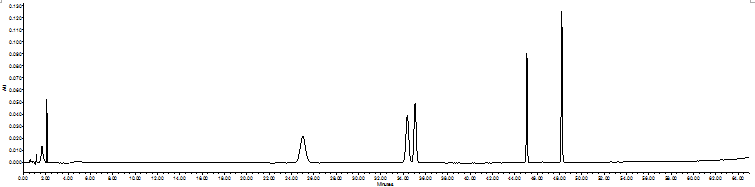


**Supplementary Figure 1**

**HPLC profiles of five reference compounds *i.e.* notoginsenoside R_1_, and ginsenosides Rg_1_, Re, Rb_1_, Rd**

**Supplementary Table 2**

**Contents of 5 saponins (mg/g) in PTS**

| Samples | NG-R_1_ | G-Rg_1_ | G-R_e_ | G-Rb_1_ | G-R_d_ |
| --- | --- | --- | --- | --- | --- |
| PTS | 184.4 | 668.3 | 108.5 | ND | ND |


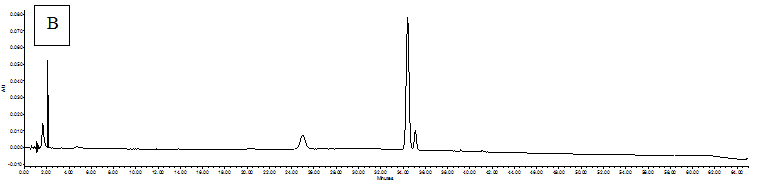


**Supplementary Figure 2**

**HPLC profile of PTS**
